# Case Report: Metachronous pancreatic adenocarcinoma following HER2-positive breast cancer and the implications of non-BRCA germline variants with TP53-mutant disease

**DOI:** 10.3389/fonc.2025.1705199

**Published:** 2025-10-28

**Authors:** Jing Chen, Hui Chun Zhou, Min Hui Fan, Mei-yao Qin

**Affiliations:** The Second Affiliated Hospital of Jiaxing University, Jiaxing, China

**Keywords:** metachronous cancers, breast cancer, pancreatic ductal adenocarcinoma, TP53 mutation, variants of uncertain significance

## Abstract

Metachronous pancreatic ductal adenocarcinoma (PDAC) following breast cancer is rare and often linked to pathogenic variants in high-penetrance genes such as BRCA2. We report a case of this clinical scenario lacking classic mutations, which prompted exploration of alternative genetic mechanisms. A 54-year-old woman was diagnosed with stage IIIB HER2-positive invasive breast cancer in 2017 and treated with neoadjuvant chemotherapy (TAC), modified radical mastectomy, radiotherapy, trastuzumab, and toremifene. Eight years later, elevated CA19–9 led to the detection of a pancreatic uncinate mass. Pathological examination after pancreaticoduodenectomy confirmed moderately differentiated PDAC. Germline testing revealed no BRCA1, BRCA2, PALB2, or ATM mutations but identified several variants of uncertain significance (VUS) in SIL1, SNX14, and ALOX12B. A somatic TP53 mutation was present in both malignancies. This case highlights that a hereditary cancer phenotype can occur even without classic mutations. It suggests that VUS in genes involved in cellular stress and metabolic pathways, particularly in combination with TP53 mutations, may contribute to the development of multiple primary malignancies. Furthermore, it underscores the importance of vigilant, phenotype-driven long-term surveillance in such patients, regardless of germline testing results.

## 1 Introduction

Advances in multimodal breast cancer therapy have significantly improved survival, resulting in a growing population of long-term survivors and an increased incidence of second primary malignancies (1, 2). Metachronous PDAC after breast cancer is a rare but serious occurrence, typically linked to hereditary predisposition syndromes involving pathogenic variants in homologous recombination repair (HRR) genes such as BRCA2, PALB2, and ATM (3–5). Identifying such mutations not only clarifies etiology but also guides treatment, potentially indicating platinum-based chemotherapy or PARP inhibitors (6).

HER2-positive breast cancer is often considered more sporadic than triple-negative or familial hormone receptor-positive subtypes, reducing suspicion for a strong hereditary component (7). Thus, the occurrence of a second primary malignancy in these patients, particularly without classic high-penetrance mutations, poses significant diagnostic and etiological challenges. Most literature and clinical guidelines focus on scenarios where a clear high-penetrance germline mutation is identified. However, a significant research gap exists in understanding the genetic drivers and optimal management of patients who present with a clear phenotype of multiple primary malignancies yet test negative for all classic hereditary cancer syndromes. This case addresses this gap by evaluating alternative explanations, such as VUS, somatic drivers, or treatment-related effects.

We present a case of HER2-positive breast cancer followed eight years later by PDAC, absent typical HRR mutations but with germline VUS and recurrent somatic TP53 mutation. The uniqueness of this case lies in the specific combination of HER2-positive breast cancer, the development of PDAC, the absence of canonical hereditary mutations, and the finding of multiple VUS in conjunction with somatic TP53 mutations in both tumors. This case illustrates the multifactorial etiology of multiple primary cancers and emphasizes the importance of integrating broader genetic and clinical perspectives beyond conventional genotype-phenotype correlations.

## 2 Case description

A 54-year-old, perimenopausal female presented to our hospital in June 2017. The patient is of Han Chinese ethnicity and worked as an office clerk prior to retirement. She reported a six-month history of a progressively enlarging, painless mass in her left breast, which she first discovered during self-examination. Other than the palpable mass, she had no complaints of breast pain, nipple discharge, or skin changes. She had no constitutional symptoms such as fever or significant weight loss. Her medical history was significant for hypertension, managed with oral antihypertensive agents, and a hysterectomy performed five years prior for uterine fibroids. She had no history of smoking or significant alcohol use. There were no known environmental or occupational exposures to carcinogens. A comprehensive family history was obtained, which revealed no malignancies in her first-degree relatives (parents, siblings, or children) or second-degree relatives. Psychosocially, the patient lived with her husband and reported a good support system. She expressed significant anxiety upon her initial cancer diagnosis but engaged well with the healthcare team.

**Figure 1:**
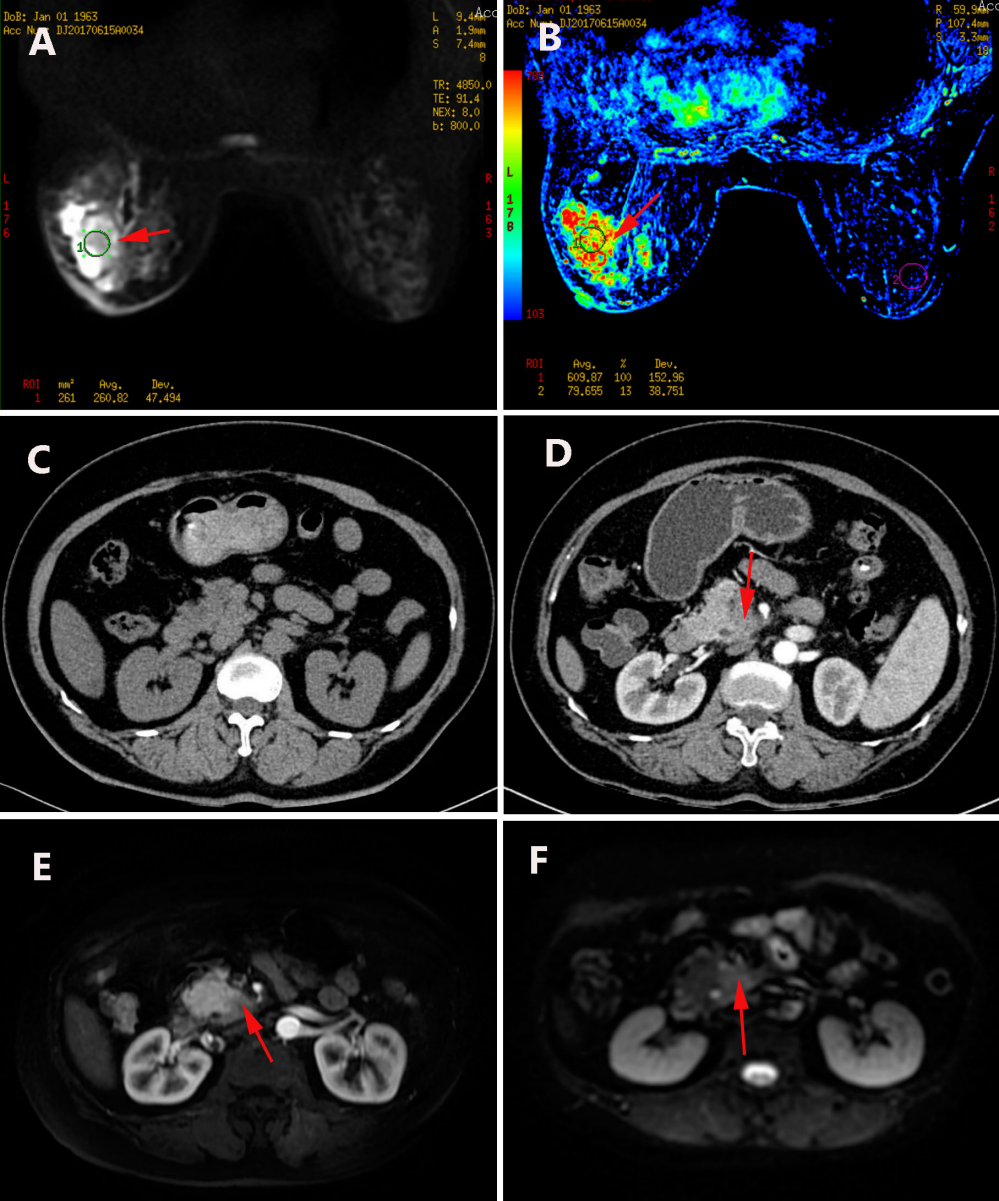
**(A)** Breast MRI demonstrates restricted diffusion in the left breast. **(B)** Parametric color map of the breast MRI. **(C)** Upper abdominal CT performed in 2017 shows no pancreatic abnormalities. **(D)** Upper abdominal CT in 2025 reveals a mass in the uncinate process of the pancreas. **(E)** Arterial phase of pancreatic MRI shows a hypodense lesion in the uncinate process. **(F)** Pancreatic MRI indicates restricted diffusion within the uncinate process. (The red arrow indicates the location of the tumor).

**Figure 2:**
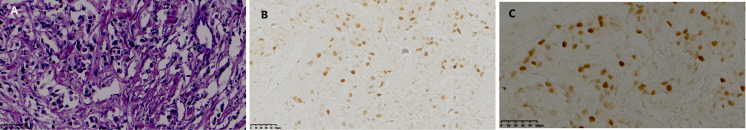
**(A)** Hematoxylin-eosin (HE) staining of the breast biopsy specimen demonstrates invasive ductal carcinoma. **(B)** Immunohistochemical (IHC) analysis revealed strong positivity for estrogen receptor (ER, 3+). **(C)** Progesterone receptor (PR) expression was also positive (2+). (The magnification is 400x).

**Figure 3:**
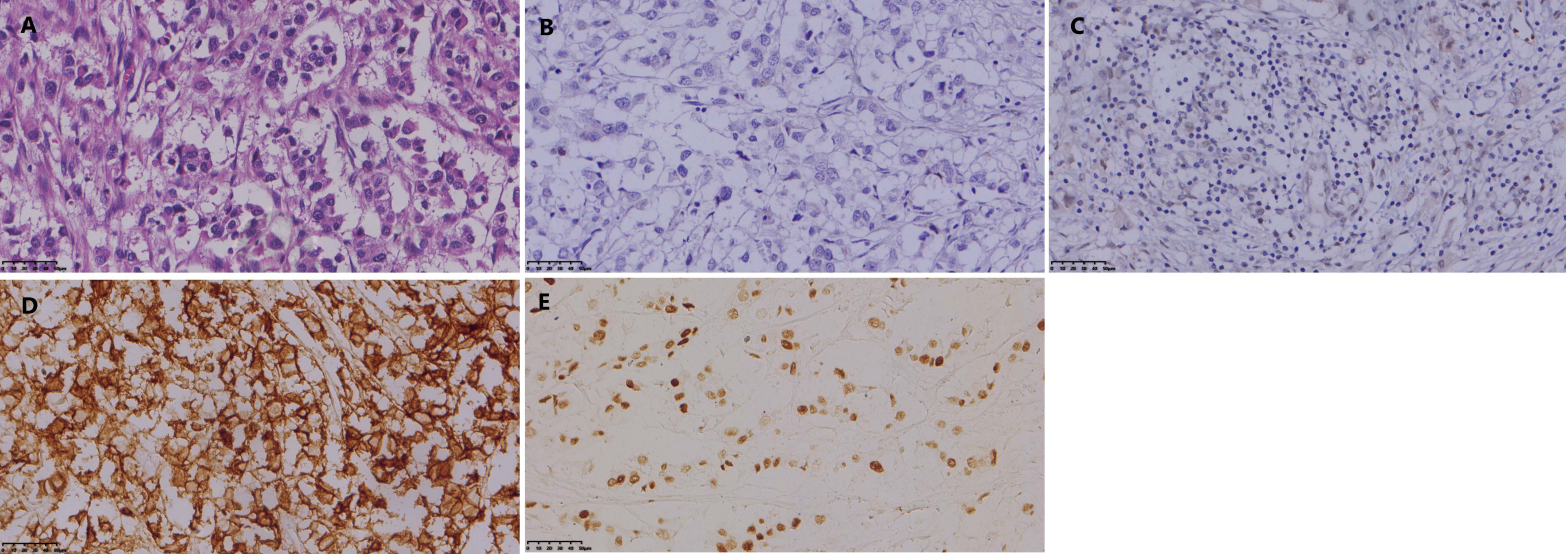
Histopathological and immunohistochemical features of the breast carcinoma resection specimen after neoadjuvant chemotherapy. **(A)** Hematoxylin and eosin (H&E) staining confirming invasive ductal carcinoma. **(B)** Complete loss of estrogen receptor (ER) expression compared to the pre-treatment biopsy. **(C)** Complete loss of progesterone receptor (PR) expression. **(D)** Retained HER2 overexpression (score 3+). **(E)** P53 protein overexpression, consistent with a TP53 mutation. (All magnifications 400x).

Physical examination revealed a palpable mass in the upper outer quadrant of the left breast with associated ipsilateral axillary lymphadenopathy. Initial diagnostic work-up included a breast ultrasound, which identified a heterogeneous mass with microcalcifications, classified as BI-RADS 5. A subsequent breast MRI confirmed an enhancing mass in the left upper outer quadrant with associated skin thickening and multiple enlarged axillary lymph nodes, highly suggestive of malignancy (**Figure 1**). An ultrasound-guided core needle biopsy of the breast mass was performed on June 14, 2017. Histopathological examination confirmed invasive ductal carcinoma. Staging examinations determined the clinical stage to be T3N2M0 (Stage IIIB). Immunohistochemical (IHC) analysis of the biopsy specimen revealed estrogen receptor (ER) positivity (3+, 40%), progesterone receptor (PR) positivity (2+, 40%) (**Figure 2**). Fluorescence *in situ* hybridization (FISH) assay demonstrated HER-2 gene amplification with a HER2/CEP17 ratio >2. Following multidisciplinary discussion, the patient received three cycles of neoadjuvant chemotherapy with the TAC regimen (Docetaxel 129 mg, Epirubicin 100 mg, and Cyclophosphamide 850 mg on day 1, every 21 days). She subsequently underwent a left modified radical mastectomy on August 15, 2017. Final surgical pathology confirmed an invasive carcinoma measuring 6.0 × 5.0 × 4.0 cm, with metastasis in 4 of 14 axillary lymph nodes. Post-chemotherapy IHC profiling showed a complete loss of hormone receptor expression (ER-, PR-) with retained HER2 overexpression (3+). Notably, the tumor exhibited P53 protein overexpression (**Figure 3**). The patient completed comprehensive adjuvant therapy, which included three additional cycles of TAC chemotherapy, 25 fractions of radiotherapy, and one year of targeted therapy with trastuzumab. Adjuvant endocrine therapy with toremifene was initiated but ultimately held given the ER-negative status on surgical specimen. The patient tolerated the treatment regimen well, with the main side effects being manageable fatigue and neutropenia. She achieved radiological and clinical remission and was placed on regular surveillance.

**Figure 4:**
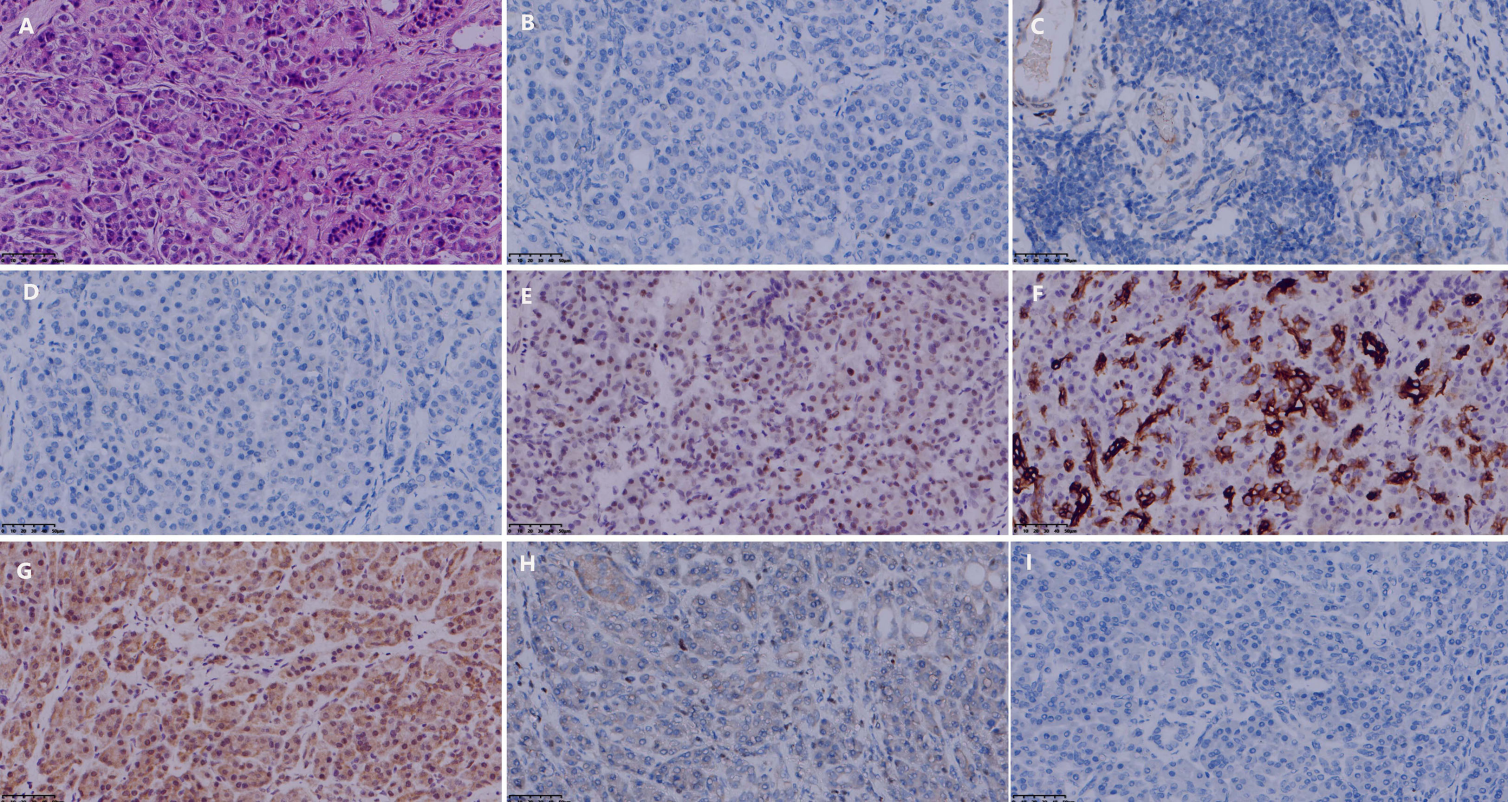
Histopathological and immunohistochemical features of the pancreatic ductal adenocarcinoma (PDAC) resection specimen. **(A)** H&E staining showing moderately differentiated ductal adenocarcinoma. **(B)** Negative estrogen receptor (ER) expression. **(C)** Negative progesterone receptor (PR) expression. **(D)** Absence of HER2 overexpression. **(E)** P53 protein overexpression, indicating a TP53 mutation. **(F)** Positive CK7 expression, supporting pancreatobiliary origin. **(G)** Retained DPC4 (SMAD4) expression. **(H)** Negative GATA3 staining, arguing against breast origin. **(I)** Negative GCDFP-15 staining, further excluding metastatic breast cancer. (All magnifications 400x).

The patient remained under regular surveillance. Eight years following her initial diagnosis, a routine follow-up in 2025 revealed an elevated serum CA19–9 level of 83.06 U/mL. The patient was asymptomatic at this time and reported no abdominal pain, jaundice, weight loss, or changes in bowel habits. Contrast-enhanced abdominal MRI and CT scans were promptly performed, identifying a suspicious 2.0 cm mass in the uncinate process of the pancreas (**Figure 1**). There was no radiological evidence of local invasion or distant metastasis. After a thorough multidisciplinary evaluation, the patient underwent a radical pancreaticoduodenectomy (Whipple procedure) on July 16, 2025. The procedure was completed successfully without intraoperative complications. Histopathological examination of the surgical specimen confirmed a moderately differentiated PDAC, measuring 2.0 × 2.0 × 1.0 cm, with presence of perineural invasion. IHC staining was crucial for confirming primary pancreatic origin: the tumor cells were diffusely positive for CK7, CK19, DPC4 (SMAD4), and MUC1, and exhibited a mutant-type pattern of P53 expression. Critically, stains for breast-specific markers GATA3 and GCDFP-15 were negative, effectively excluding metastatic breast disease (**Figure 4**).

Given the clinical history of two primary malignancies, germline genetic testing via whole-exome sequencing was pursued. The analysis identified no pathogenic or likely pathogenic variants in the BRCA1, BRCA2, PALB2, or ATM genes. Specifically, the BRCA1 and BRCA2 genes were fully analyzed, and no mutations were detected. However, it revealed several VUS in genes including SIL1, SNX14, ALOX12B, MPIG6B, and TMC6 (**Table 1**). The patient’s postoperative recovery was uneventful. Her case was reviewed at a multidisciplinary tumor board, and adjuvant chemotherapy with gemcitabine and albumin-bound paclitaxel was recommended. Genetic counseling was provided to discuss the implications of the VUS findings for the patient and her family.

**Table 1:** Germline variants of uncertain significance (VUS) identified by whole-exome sequencing.

Gene	Chromosome coordinate	Variant information	Gene subregion
SIL1	chr5: 139121064	NM_022464.5: c.215del: p.Pro72ArgfsTer 22	Exon 3/10
SNX14	chr6: 85565361- 85565362	NM_153816.6: c.519dup: p.Ala174CysfsTer 34	Exon 6/29
ALOX12B	chr17: 8079525- 8079526	NM_001139.3: c.938_941dup: p.Ala316ProfsTer 59	Exon 8/15
MPIG6B	chr6: 31723930- 31723934	NM_138272.3: c.354_358del: p.Val119SerfsTer 59	Exon 2/6
TMC6	chr17: 78119009	NM_001127198. 5: c.1849C>T: p.Gln617Ter	Exon 15/20

## 3 Discussion

### 3.1 Diagnosis of multiple primary malignancies

The diagnosis of metachronous multiple primary malignancies (MPMs) in this case was rigorously established in accordance with the Warren and Gates criteria, which mandate that each tumor display definitive malignant histology, arise from distinct anatomical sites, and that metastatic spread be definitively excluded (8). Histopathological assessment revealed fundamentally divergent morphologies: the breast tumor presented as an invasive carcinoma of no special type, whereas the pancreatic tumor exhibited gland-forming structures typical of a moderately differentiated ductal adenocarcinoma. Immunohistochemical analysis provided key discriminatory evidence: the pancreatic tumor showed diffuse positivity for CK7, CK19, and DPC4 (SMAD4), and was entirely negative for the breast-specific markers GATA3 and GCDFP-15 (9, 10). This immunoprofile is consistent with a primary pancreatobiliary origin and effectively excludes metastatic breast disease. Additionally, the stark contrast in treatment response and biomarker status, particularly the complete loss of hormone receptor expression (ER/PR) in the breast tumor following neoadjuvant chemotherapy, further supports their biological independence. Collectively, these histopathological, immunohistochemical, and clinical features provide compelling evidence that the two tumors represent distinct primary malignancies, each with an independent clonal origin.

The observed transition from ER/PR positivity in the pre-treatment biopsy to negativity in the post-neoadjuvant resection specimen is a well-recognized example of clonal evolution under therapeutic selection pressure. This shift is not an artifact of diagnostic interpretation but rather reflects the potent selective impact of cytotoxic chemotherapy (11, 12). The TAC regimen (docetaxel, epirubicin, and cyclophosphamide) preferentially targets rapidly dividing cells, particularly the hormone receptor-positive and chemotherapy-sensitive subpopulation. Consequently, this susceptible clone is effectively eradicated, while pre-existing or adaptively resistant hormone receptor-negative tumor cells, which may employ alternative survival pathways, persist, expand, and ultimately dominate the residual disease (13, 14). This mechanism is further reinforced by the concomitant emergence of P53 protein overexpression in the residual tumor, a feature frequently associated with genomic instability and more aggressive, treatment-resistant phenotypes (15–17). Thus, the complete loss of ER/PR expression underscores the dynamic heterogeneity and adaptive potential of the tumor, with significant implications for subsequent therapeutic decisions, necessitating a shift from endocrine therapy toward alternative strategies such as anti-HER2 agents.

### 3.2 Exploring the genetic basis and the role of VUS and TP53

The presentation of early-onset, node-positive breast cancer followed by a second primary tumor raised substantial concern for an underlying genetic predisposition. Pathogenic variants in high-penetrance genes such as BRCA2 (strongly associated with both breast and pancreatic cancer), PALB2 (a functional partner of BRCA2), and TP53 (linked to Li-Fraumeni syndrome) were considered primary candidates (18–20). Comprehensive germline testing, which included full analysis of the BRCA1 and BRCA2 genes, identified no pathogenic variants in these or other classic high-penetrance genes. The observed P53 overexpression in the breast cancer and mutant-pattern P53 expression in the pancreatic carcinoma further suggested a potential shared genetic etiology. However, the absence of pathogenic germline mutations in BRCA1, BRCA2, PALB2, and ATM redirected the etiological focus. The absence of any family history of cancer in first- and second-degree relatives, while reducing the likelihood of a highly penetrant autosomal dominant syndrome, does not entirely rule out genetic factors, as *de novo* mutations or incomplete penetrance can occur.

In the absence of a clear monogenic cause, several alternative explanations must be considered. The identification of VUS in genes including SIL1 (involved in endoplasmic reticulum stress response), SNX14 (associated with lipid metabolism and autophagy), and ALOX12B (implicated in inflammatory signaling) is particularly notable (21–23). First, these VUS could be benign “passenger” variants with no real clinical significance. Second, an undetected high-penetrance variant or a complex structural variant missed by the testing methodology could be present. Third, non-genetic factors, such as prior therapy, environmental exposures, or lifestyle, may have contributed, though the patient had no notable exposures beyond her documented chemotherapy. Finally, a polygenic model of risk, where the cumulative effect of multiple low-to-moderate risk alleles lowers the threshold for carcinogenesis, is plausible. While the individual contribution of each VUS to cancer risk remains uncertain, it is plausible that their cumulative effect established a genetic background of enhanced cellular instability, a “fertile ground” that considerably lowered the threshold for carcinogenesis. This oligogenic or polygenic risk model is increasingly accepted in oncology to explain cancer predisposition in cases lacking a conventional monogenic driver (24). In this patient, the polygenic model is considered a plausible, though speculative, mechanism that could be explored in future research through functional assays of the identified VUS or polygenic risk score analysis in larger cohorts.

A central unifying molecular feature across both malignancies was aberrant TP53 pathway activity. The breast cancer specimen showed significant P53 protein overexpression, a common immunohistochemical surrogate for TP53 mutation, while the pancreatic adenocarcinoma unequivocally exhibited a mutant-type pattern of P53 expression. Somatic TP53 mutation is a well-established marker of genomic instability and a pivotal late-stage driver event in many cancers (25, 26). Its prominence in two independent primary tumors suggests that it acted synergistically, accelerating the process of carcinogenesis initiated by the patient’s underlying germline susceptibility, potentially influenced by the cumulative effect of the identified VUS or other unidentified factors. This pattern underscores that concurrent TP53 dysfunction likely represents a critical molecular convergence point, rather than a stochastic event, facilitating tumor development across different tissues through disruption of fundamental cellular integrity and DNA damage response mechanisms.

### 3.3 Clinical management and surveillance implications

The management of metachronous malignancies necessitates an individualized, multidisciplinary approach that integrates tumor-specific therapies while accounting for cumulative toxicities from prior treatments (27). In this patient, the breast cancer was managed with standard multimodal therapy, achieving prolonged remission. The subsequent pancreatic cancer was treated with curative-intent surgery, illustrating that prior exposure to anthracycline- and taxane-based chemotherapy did not preclude major abdominal surgery. In the absence of targetable germline mutations, adjuvant treatment for pancreatic cancer was guided toward conventional options (gemcitabine-based regimens), rather than platinum or PARP inhibitor strategies (28). This case underscores that a history of multiple primary tumors calls for lifelong, tailored surveillance and highlights the importance of balancing curative intent against cumulative therapeutic risks.

### 3.4 Clinical implications and impact in resource-limited settings

The diagnosis of this condition has profound clinical implications for both the practitioner and the patient. For clinicians, especially in resource-limited settings where comprehensive genetic testing may not be readily available, this case underscores that a phenotype of multiple primary cancers should itself be considered a strong indicator of underlying cancer predisposition. Even in the absence of a confirmed mutation, such patients warrant heightened, lifelong surveillance. Practitioners should prioritize a thorough personal and family history and maintain a high index of suspicion for second malignancies. The key diagnostic takeaway is the critical importance of immunohistochemistry in differentiating a new primary from a metastasis, as this directly determines management, curative-intent surgery for a new primary versus systemic therapy for a metastasis. In this case, the negative GATA3/GCDFP-15 and positive pancreatobiliary profile in the pancreatic tumor were pivotal.

For the patient, the impact is multifaceted. Firstly, receiving a diagnosis of a second, independent cancer is psychologically challenging and necessitates robust psychosocial support. Secondly, it implies a lifelong commitment to rigorous, tailored cancer surveillance, which can be both financially and emotionally burdensome. However, the positive impact is the opportunity for early detection and curative treatment, as demonstrated here where the pancreatic cancer was detected at a resectable stage. Finally, the identification of VUS, while currently of uncertain significance, provides a potential explanation for their condition and a framework for future research, which can be empowering for some patients.

### 3.5 Study limitations and future directions

This study has several limitations. The primary limitation is the inherent difficulty in proving causality for the identified VUS. Without functional studies or segregation analysis in affected family members, which was not possible given the negative family history, their role remains hypothetical. The BRCA1 and BRCA2 genes were comprehensively analyzed, but we cannot entirely exclude the presence of other undetected pathogenic variants in non-coding regions or genes not covered by the testing panel. Furthermore, the contribution of prior therapeutic exposures to the development of the second malignancy, while considered, is difficult to quantify. Future research should focus on functional characterization of VUS in genes like SIL1, SNX14, and ALOX12B to elucidate their potential role in cancer pathways. Additionally, aggregating similar cases through multi-institutional collaborations will be crucial to validate the proposed oligogenic risk model.

## 4 Conclusion

This case of metachronous HER2-positive breast cancer and PDAC, lacking classic high-penetrance mutations, illustrates complex polygenic predisposition. Chemotherapy-induced receptor loss exemplifies clonal evolution, while TP53 mutation and germline VUS suggest synergistic roles in genomic instability. The findings emphasize phenotype-driven, long-term surveillance and the integration of molecular profiling with clinical assessment for optimal management of multiple primary malignancies.

## Data Availability

The original contributions presented in the study are included in the article/supplementary material. Further inquiries can be directed to the corresponding author.
